# Effects of season and single layer centrifugation on bull sperm quality in Thailand

**DOI:** 10.5713/ajas.19.0624

**Published:** 2019-12-24

**Authors:** Thanapol Nongbua, Apirak Utta, Nutthee Am-in, Junpen Suwimonteerabutr, Anders Johannisson, Jane M Morrell

**Affiliations:** 1 Division of Reproduction, Department of Clinical Sciences, Swedish University of Agricultural Sciences (SLU), Box 7054, Uppsala 75007, Sweden; 2 Faculty of Veterinary Sciences, Mahasarakham University, Maha Sarakham 44000, Thailand; 3 North-Eastern Bull Center, Bureau of Biotechnology in Livestock Production, DLD, Khon Kaen 40000, Thailand; 4 Department of Obstetrics, Gynaecology and Reproduction, Faculty of Veterinary Science, Chulalongkorn University, Bangkok 10330, Thailand

**Keywords:** Temperature, Humidity, Bovine Semen Quality, Colloid Centrifugation, Frozen Semen

## Abstract

**Objective:**

The aim of study was to investigate the effects of season and single layer centrifugation (SLC) before cryopreservation on post-thaw bull sperm quality in Thailand.

**Methods:**

Semen was collected from 6 bulls (*Bos indicus*) in summer, rainy season and winter 2014 through 2016. Semen characteristics, sperm morphology, sperm kinematics, viability, chromatin structure and mitochondrial membrane were evaluated. Meteorological data were available from the local meteorological station;

**Results:**

Season had an effect on semen characteristics in the raw ejaculate, with higher proportions of normal spermatozoa and lower abnormalities in winter than in the other two seasons. Sperm kinematics, viability, DNA fragmentation index, and mitochondrial membrane potential were not different between seasons. Sperm samples selected by SLC had greater normal morphology and a lower proportion with bent tails than controls and higher values of progressive motility (PRO), beat cross frequency, linearity, straightness, wobble (WOB), and lower values of slow motility, velocity average path (VAP), velocity curved line, and amplitude of lateral head displacement than controls. In addition, SLC-selection had a favorable effect on PRO, VAP, and WOB that differed among seasons.

**Conclusion:**

Our results suggested that these bulls were well adapted to their location, with season having an effect on sperm morphology. Moreover, SLC could be used prior to cryopreservation, regardless of season, to enhance normal morphology and kinematics of bull sperm samples without adversely affecting other parameters of sperm quality. However, there was considerable variation among bulls in DNA fragmentation index, mitochondrial membrane potential and sperm viability. In addition, SLC had a positive effect on sperm morphology and sperm kinematics, which could be expected to influence fertility.

## INTRODUCTION

Many factors, including genetics and environment, can affect semen quality [1]. Season is one of these factors, as reported in many studies [2–4]. Season has an impact on reproductive performance through macro- and micro-climatic factors such as temperature, humidity, rainfall and photo-period [5,6] and may depend on geographic location. However, the reported effects of season are not always consistent. The sperm quality of bulls housed in northern Spain was affected by season, with sperm quality being better during spring than in other seasons [2]. In contrast, a study in Brazil showed that ambient temperature, humidity and season did not affect sperm production and semen quality [7]. Breed can also affect sperm quality in different seasons. Studies in *Bos taurus* showed that ejaculate volume and total sperm differed among seasons in different breeds [1] whereas a study in southeastern Brazil showed that ejaculate volume, sperm concentration, gross-motility, progressive sperm motility, vigor and morphological sperm defects were significantly affected by season and genotype between *Bos indicus* and *Bos taurus* [8].

Independently of any seasonal effect, bull sperm quality may need to be improved for artificial insemination (AI). Colloid centrifugation has been used to improve sperm quality in different species, especially single layer centrifugation (SLC) using one layer of a species-specific colloid, e.g. stallion [9], bull [10]. This method has been shown to improve bull sperm quality [11,12].

Thailand is located in South East Asia and has a tropical climate. A seasonal effect on sperm quality has been reported in swamp buffalo [13–16] and boar [17,18] but not in bull in Thailand. The objective of the present study, therefore, was to investigate the effects of season, and SLC before cryopreservation, on post-thaw bull sperm quality in Thailand.

## MATERIALS AND METHODS

### Bovine semen collection and preparation

The collection of the material needed for this study did not require ethical approval in Thailand or Sweden. The animals were housed and kept under national and international guidelines. The animals were privately owned by the breeding station; permission was granted by the breeding station to use the ejaculates they provided for research purposes.

Semen was collected from 6 *Bos indicus* bulls: American Bhraman (n = 4) and Sahiwal (SW; n = 2), which were used for routine semen collection at the North Eastern bull center, Department of Livestock Development, Khon Kaen, Thailand. Their body condition score on a scale of 1 to 5 was 3.50 to 3.75. The age of bulls at the start of semen collection was 8.0±2.60 years (mean±standard deviation) range 4 to 11 years). The bulls were fed on grass (*Panicum maximum* and *Brachiaria ruziziensis*), commercial concentrate and minerals supplement. They were housed in an open barn, as described previously [16].

### Semen extender

The semen was extended in egg yolk tris medium, consisting of 30.28 g tris(hydroxymethyl)aminomethane (tris), 17 g citric acid, 12.5 g fructose, 80 mL glycerol and 920 mL deionised water, with egg yolk (250 mL), penicillin (1,000,000 international units) and streptomycin (1,000 mg). The chemicals were reagent grade, purchased from Sigma.

### Metereological data

Semen was collected in three seasons: summer (May to June 2014), rainy season (September to October 2015), and winter (January to February 2016). Data on the ambient temperature (°C), humidity (%), and rainfall (mm) for the study period were accessed from the North Eastern Meteorological center (upper part), Khon Kaen, Thailand, which is located adjacent to the bull center [16]. The meteorological data were used to calculate a thermal humidity index (THI) following a standard formula from the National Research Council [19], as follows:

THI=(1.8×T+32)-[(0.55-0.0055×RH)×(1.8×T-26)],

Where T = temperature and RH = relative humidity.

### Experiment 1

**Figure 1:**
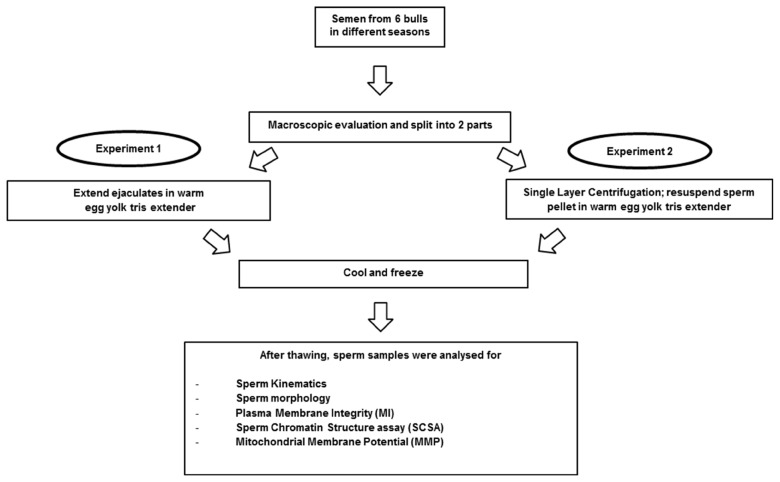
Experimental design.

Semen was collected approximately once per week according to routine husbandry procedures, using the first ejaculation of each bull. Macroscopic evaluation was carried out as follows: volume, concentration (Spectro 22RS, LaboMed, Inc., Los Angeles, CA, USA; million/mL), pH (IQ Scientific ISFET Handheld pH/mV Meter; IQ Science Instruments, Col-Parmer, IL, USA) and subjective sperm motility before processing (%MO) (Figure 1). Subsequently the ejaculate was extended in warm (35°C) egg yolk tris medium to achieve a final sperm concentration of 80×10^6^ spermatozoa/mL. The sperm samples were cooled to 4°C over 4 h, packed into straws and frozen according to the protocol at the semen center. The straws were kept in liquid nitrogen until thawing at 37°C for 12 seconds for evaluation. Sperm kinematics, sperm morphology, membrane integrity (MI), chromatin integrity and mitochondrial membrane potential (MMP) were assessed.

### Experiment 2

After macroscopic evaluation, the ejaculate was split: one part (control) was prepared following the standard procedure at the bull center. The other part was used for SLC with Bovicoll [20] and was then frozen using the same protocol as control (Figure 1). The SLC with Bovicoll (patent applied for, J. M. Morrell), was carried out according to Nongbua et al [11]. The ejaculate was extended in warm (35°C) egg yolk tris medium (50×10^6^ spermatozoa/mL) and 15 mL was layered over 15 mL of Bovicoll in a 50 mL conical centrifuge tube. The preparation was centrifuged at 300×*g* for 20 min at room temperature. After centrifugation, the supernatant was aspirated, the sperm pellet was harvested from beneath the last 1 to 2 mL colloid and resuspended in egg yolk tris medium. Freezing, thawing and post-thaw analyses were performed as for experiment 1.

### Sperm morphology

**Figure 2:**
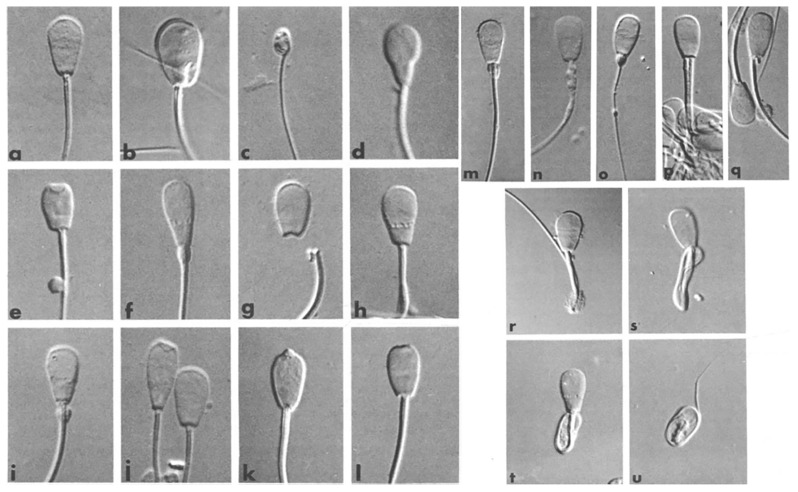
Contrast micrographs (differential interference) of fixed bull spermatozoa from wet preparations (×1,900 magnification). a: normal head; b. giant head with abaxial midpiece; c. underdeveloped head; d. pear shaped head; e. short head with knobbed acrosome and distal cytoplasmic droplet; f. tapered head with “diadem” defect and proximal cytoplasmatic droplet; g, Detached heads; h. nuclear pouches (“diadem” defect), i. nuclear pouches; j. tapered head with knobbed acrosome + spermatozoa with normal head shape, k. knobbed acrosome, 1. flat acrosome; m, abnormal midpiece; n. defective midpiece with mitochondria1 disarray; o. filiform midpiece; p, double tail; q, accessory vestigial midpiece; r, single bent tail with distal cytoplasmic droplet; s, double folded tail; t, coiled tail under the head; u. coiled tail around the head, possibly underdeveloped (modified from [23]).

After thawing, an aliquot of each sample was fixed in formal saline and a further aliquot (10 μL) was used for the preparation of an air-dried smear. The fixed samples were used for evaluation of 200 spermatozoa in wet preparations [21] using a phase-contrast microscope (Leica Microsystems, Wetzlar, Germany) at 1,000× magnification. The air-dried smears were stained with carbolfuchsin-eosin according to Williams [22] for evaluation of head shape in 500 spermatozoa. Sperm morphology was classified as described by Söderquist [23] as shown in Figure 2. The proportion of abnormal spermatozoa was subtracted from 100 to give the mean proportion of morphologically normal spermatozoa. All samples were evaluated by skilled personnel in the Swedish Sperm Reference Laboratory, Swedish University of Agricultural Sciences [24].

### Computer assisted sperm analysis

Motility analysis was performed on (5 μL) aliquots using a CEROS II Version 1.7 (Beverly MA, USA) connected to a microscope (Zeiss, Axiolab A1, Jena, Germany) with a heated stage (38°C). The following sperm kinematics were evaluated: total motility (a spermatozoon that moves more than its head length from its original position during the acquisition; MOT, %), progressive motility (a spermatozoon moving with STR >80 and VAP >50; PRO, %), slow motility (a spermatozoon moving with VSL <30 or VAP <20; SLOW, %), static motility (a spermatozoon moving with VSL <1 or VAP <4; STAT, %), velocity average path (VAP, μm/s), velocity curved line (VCL, μm/s), velocity straight line (VSL, μm/s), amplitude of lateral head displacement (ALH, μm), beat cross frequency (BCF; Hz), linearity (LIN, VSL/VCL; %), straightness (STR, VSL/VAP; %), and wobble (WOB, VAP/VCL; %).

### Plasma membrane integrity

Plasma MI was analysed by flow cytometry after staining with SYBR14 and propidium iodide (PI) [12]. Briefly, the samples were diluted with buffer B (patent applied for; J. M. Morrell and H. Rodriguez-Martinez) to a final concentration of 2×10^6^ sperm cells/mL (300 μL). The diluted samples were stained with 0.6 μL of 20 μM SYBR14, 3 μL of 24 mM PI (Live-Dead Sperm Viability Kit L-7011; Invitrogen, Eugene, OR, USA) and incubated at 37°C for 10 min before evaluation using a FC500 flow cytometer (Beckman Coulter, Brea, CA, USA). Excitation was obtained by an argon-ion laser (488 nm). Red fluorescence was detected via a FL3 band-pass filter (610 nm) and green fluorescence was evaluated via fluorescence channel (FL1) band-pass filter (525 nm). In total, 50,000 spermatozoa cells were analysed. After gating to include only spermatozoa, they were classified as living (%) (intact membrane, SYBR14-positive/PI-negative), dead or dying (%) (damaged membrane, SYBR14- negative/PI-positive; or SYBR14-positive/PI-positive, respectively).

### Sperm chromatin structure

Sperm chromatin integrity was evaluated according to the method of Evenson [25] with slight adaptations [14]. Briefly, the sperm samples were mixed 50:50 μL with Tris-sodium chloride-EDTA (ethylenediaminetetraacetic acid) (TNE) buffer (0.15 mol/L NaCl, 0.01 mol/L Tris-HCl, 1 mmol/L EDTA, pH 7.4) and snap-frozen in liquid nitrogen vapor before storage at −80°C. The samples were thawed on ice approximately 20 minutes before analysis and 10 μL were diluted with 90 μL of TNE buffer. Partial DNA denaturation *in situ* was performed by mixing with 0.2 mL of a low pH detergent solution containing 0.17% Triton X-100 (0.15 mol/L NaCl, and 0.08 mol/L HCl; pH 1.2). After 30 s the denatured sperm were stained with 0.6 mL of acridine orange (6 μg/mL in 0.1 mol/L citric acid, 0.2 mol/L Na_2_HPO_4_, 1 mmol/L EDTA, 0.15 mol/L NaCl; pH 6.0) and were evaluated by flow cytometry within 5 minutes of acridine orange staining. The standard optical equipment of a FC500 flow cytometer (Beckman Coulter, USA) was used and forward scatter, side scatter, green (FL1, 525 nm band-pass filter) and red (FL3, 610 nm band-pass filter) fluorescence for 10,000 cells was collected. A gate restricting the analysis to spermatozoa was placed in the FSC-SSC dot-plot. The data were analysed using FCS express version 2 (Denovo Software, Thornhill, ON, Canada) to calculate the DNA fragmentation index (%DFI) by placing regions in the histogram of the alpha-t-distribution (alpha-t = red/red+green fluorescence) for each analysed spermatozoon.

### Mitochondrial membrane potential

The MMP of sperm cells was evaluated using the cationic probe 5,5′,6,6′-tetrachloro-1,1′,3,3′-tetraethyl-benzimidazolylcarbocyanine iodide (JC-1) [12,26]. Briefly, the samples were diluted to a final concentration 2.5×10^6^ sperm cells/mL with buffer B. The diluted samples were stained with 1.2 μL of 3 mM JC-1 and incubated at 37°C for 40 min. After incubation, the stained samples were analysed with a FC500 flow cytometer (Beckman Coulter, USA) using an argon-ion laser (488 nm). Emitted fluorescence was collected using both FL1 (525 nm) and FL2 (575 nm) filters. Green fluorescence was analysed in FL1 and orange in FL2, with compensation between these parameters. Spermatozoa were gated on the FSC-SSC dot-plot and 30,000 cells were classified as having high respiratory activity (%) (orange fluorescence) or low respiratory activity (%) (green fluorescence).

### Statistical analysis

In all response variables, residuals were examined for normality and homoscedasticity using diagnostic plots. The data analysis was performed using the mixed procedure of SAS, version 9.3 (Proc Mixed, SAS, Cary, NC, USA).

The effects of meteorological data was analysed as follows; the fixed part of the model was of type y = seasons, x = time between season as a random factor, where y is the response variable meteorological data (temperature, humidity, and rainfall).

Experiment 1, mean values for sperm quality parameters after thawing were analysed as follows; the fixed part of the model was of type y = seasons, x = bull and interaction between season as a random factor, where y is the response variable (sperm characteristic, sperm morphology after thawing and sperm quality after thawing).

Experiment 2, the effects of SLC in different seasons on sperm quality after thawing were analysed as follows; the fixed part of the model was of type y = seasons, SLC and interaction between them, x = bull and interaction between season as a random factor, where y is the response variable (sperm morphology after thawing and sperm quality after thawing).

Post-hoc comparisons were adjusted for multiplicity using Tukey’s method in the both experiments. All values are presented as least squares means±standard error of the mean. A p value of <0.05 was considered statistically significant.

## RESULTS

### Meteorological data

Temperature was significantly different between the seasons (p<0.001). The highest temperature occurred in summer (29.7 ±0.5) and differed from rainy season (27.6±0.5) (p<0.05) and winter (25.4±0.5) (p<0.001). The temperature was higher in the rainy season than in winter (p<0.05). The humidity also differed significantly between seasons (p<0.05). The rainy season (80.9±1.8) had the highest humidity and differed significantly from winter (73.6±2.4) (p<0.05) but there was no significant difference between the rainy season and summer (78.3±2.9). Furthermore, there was a trend for rainfall to be higher in the rainy season (151.4±34.3) than in winter (17.4 ±34.3) (p = 0.05) but there was no significant difference in rainfall between summer (79.4±34.3) and winter.

The THI was significantly different between the seasons (p<0.01). The lowest THI occurred in winter (74.8±1.4) and differed from the rainy season (80.5±0.8) (p<0.01) and summer (82.7±0.5) (p<0.001). There were no significant differences in THI between the rainy season and summer (Table 1).

### Experiment 1

#### Semen characteristics

Semen characteristics in different seasons are shown in Table 1. There was a significant difference in pH among seasons (p<0.0001), being lower in winter than in the rainy season (p<0.05) and summer (p<0.0001). The pH was higher in summer than in the rainy season (p<0.01). There were no significant differences in volume, concentration or sperm subjective motility among seasons.

#### Sperm morphology

Normal morphology (Table 1) was higher in winter compared to summer (p<0.05) and the rainy season (p<0.01). Furthermore, acrosome defects were lower in winter than in the rainy season (p<0.05), proximal droplets were lower in winter than in summer (p<0.01) and bent tails were lower in winter than in the rainy season (p<0.05).

#### Sperm kinematics, plasma membrane integrity, DNA fragmentation, and mitochondrial membrane potential

There were no significant differences in sperm kinematics (Table 2), MI, DNA fragmentation (%DFI) and MMP among seasons (Table 3).

### Experiment 2

#### Sperm morphology

The SLC samples had higher normal morphology (p<0.05) and lower bent tails than controls (p<0.05) (Table 4). There were no significant differences in other sperm abnormalities between SLC and control (p>0.05), nor was there an interaction between treatment and seasons.

#### Sperm kinematics

**Figure 3:**
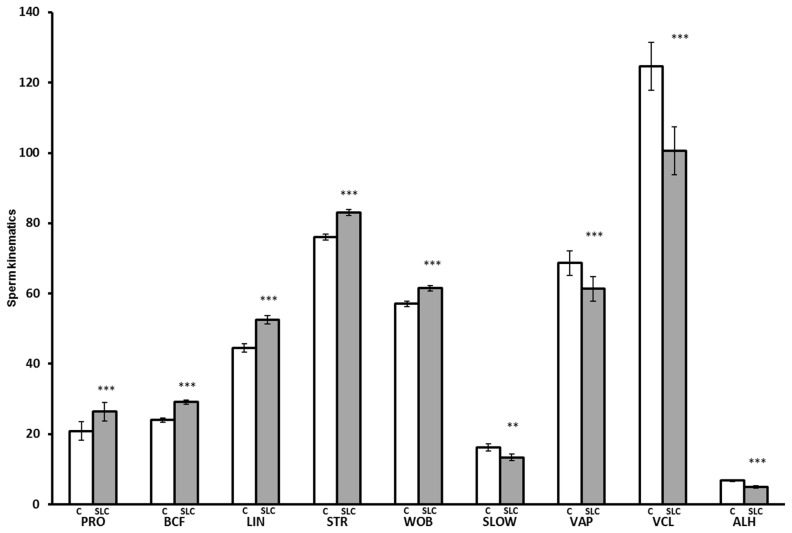
Sperm kinematics after thawing for control (C, white bar) and single layer centrifugation (SLC, gray bar) (LSMEAN±SEM) (n = 79 each for control and SLC; summer; n = 19, rainy; n = 29, and winter; n = 31). LSMEAN±SEM, least squares means±standard error of the mean; PRO, progressive motility (%); BCF, beat cross frequency (Hz); LIN, linearity (VSL/VCL, %); STR, straightness (VSL/VAP, %); WOB, wobble (VAP/VCL, %), SLOW, slow motility (%); VAP, velocity average path (μm/s); VCL, velocity curved line (μm/s); ALH, amplitude of lateral head displacement (μm). ** p<0.01, *** p<0.001.

**Figure 4:**
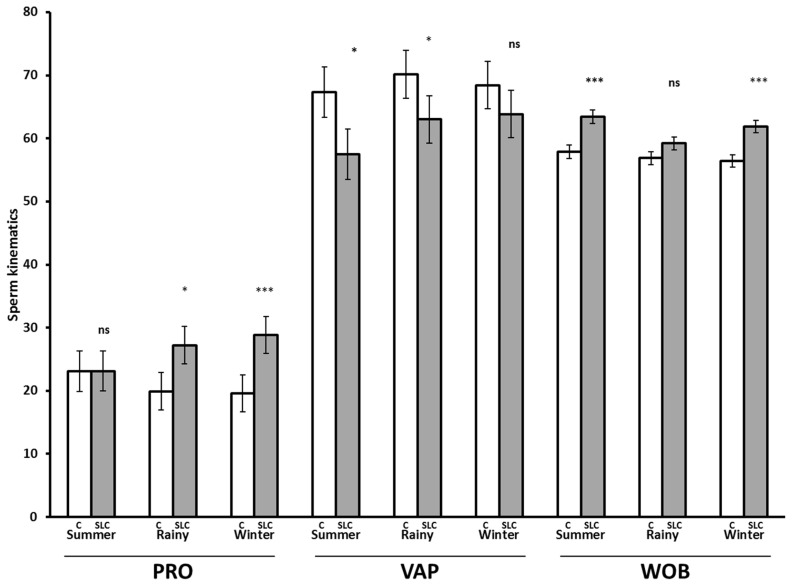
Post-thaw sperm kinematics for control (C, white bar) and single layer centrifugation (SLC, gray bar) in different seasons (LSMEAN±SEM) (n = 79 each for control and SLC; summer, n = 19; rainy, n = 29; and winter, n = 31). LSMEAN±SEM, least squares means±standard error of the mean; PRO, progressive motility (%); VAP, velocity average path (μm/s); and WOB, wobble (VAP/VCL, %). ns, no significant difference; * p<0.05, *** p<0.001.

The SLC samples showed greater values for PRO (p<0.001), BCF (p<0.0001), LIN (p<0.0001), STR (p<0.001), and WOB (p<0.001) than control, whereas they showed lower values for SLOW (p<0.01), VAP (p<0.0001), VCL (p<0.0001), and ALH (p<0.0001) than control (Figure 3). There were no significant differences in MOT and STAT between SLC and control (p>0.05). The interaction between the season and treatment was significant for PRO (p<0.05), VAP (p<0.05), and WOB (p<0.05). The SLC-samples had a greater PRO than control in the rainy season (p<0.05) and winter (p<0.0001) but were not different from control in summer (p>0.05). The SLC had a lower VAP than control in summer and the rainy season (p<0.05) but there was no effect of treatment in winter. The SLC samples had higher WOB than control in summer and winter, although there was no significant effect on WOB in the rainy season (Figure 4).

#### Plasma membrane integrity, DNA fragmentation, and mitochondrial membrane potential

There were no significant differences in MI (living spermatozoa; 31.7±4.2, 33.4±4.2), %DFI (6.5±1.2, 5.8±1.2) or MMP (high MMP; 36.1±4.4, 36.1 ±4.7) between SLC and control respectively (p>0.05). Furthermore, there were no effects of treatment, and no interaction between season and treatment for MI (living spermatozoa, SLC and control respectively; summer, 23.4±5.1, 30.3±5.1; rainy season, 34.9±5.2, 33.3±5.2; winter, 36.7±5.0, 36.6±5.0), %DFI (summer, SLC and control respectively; summer, 6.9 ±1.8, 6.0±1.8; rainy season, 5.5±1.9, 5.7±1.9; winter, 7.0±1.8, 6.0±1.8) or MMP (high MMP, SLC and control respectively; summer, 30.5±4.5, 34.9±4.5; rainy season, 36.6±4.5, 34.4±4.5; winter, 41.2±4.4, 39.1±4.4).

## DISCUSSION

The objectives of this study were to determine the effect of season and SLC on post–thaw bull sperm quality. Previous studies on the effects of season on sperm quality vary considerably, with some studies showing a negative effect of high temperatures and humidity whereas other studies do not show effects of season on sperm quality. Thailand has a tropical climate, which might be expected to have an adverse effect on sperm quality. Sperm quality can be improved by selecting certain sub-populations with SLC; thus, one of the aims was to see if there was an interaction between season and the ability of SLC to select robust spermatozoa.

Temperature and humidity were different, and rainfall tended to be different among seasons. However, during the period of the study, there were few differences between summer and the rainy season, which may be a confounding factor in the interpretation of our results. The results of experiment 1 indicated that there were differences in semen pH and sperm morphology among seasons. There were higher proportions of normal spermatozoa and fewer morphological abnormalities in winter than in the rainy season and summer. However, sperm kinematics, viability, %DFI and MMP were not different between seasons. Although 21 months elapsed between starting sampling in summer and finishing sampling in winter, there is no suggestion that the increasing age of the bulls affected the results. The differences were seen in the rainy season, with only pH and normal morphology being different between summer and winter. These parameters were not related to the age of the bull in these individuals.

The effects of season on sperm quality have been reported in several studies. However, the factors involved, such as breed or age of bulls, the geographic locations of study, meteorological data, and species, varied in different studies. Our observations of a seasonal effect on morphology (although there was no difference in sperm kinematics, viability, %DFI and MMP with season) are in partial agreement with a study in Simmental bulls in Brazil in which a higher proportion of major sperm defects was seen during the summer than in winter; breed and season affected minor sperm defects, whereas season affected total defects [27]. However, another study in Brazil indicated that neither ambient temperature, humidity nor season affected sperm production and semen quality. Nevertheless, the genotype or age of bulls can affect total number of spermatozoa and viability, and varies in different years [7]. Our results are also in partial agreement with previous studies on swamp buffalo in Thailand, wherein ejaculate volume, pH, sperm concentration, total sperm number and initial sperm motility did not differ between seasons. However, in the latter study, MI and the proportion of morphologically normal spermatozoa were greatest in summer and lowest in winter [14], in contrast to our results. In a study in boars in Thailand, housed in either a conventional open air system or housing with an evaporative cooling system, there were minor differences in temperature and humidity between seasons, and differences in the seasonal pattern of sperm production between the two housing systems. High temperature and high humidity had unfavorable effects on sperm production [17] and on sperm morphology [18].

In contrast to our results that sperm kinematics, viability, %DFI and MMP were not different between seasons whereas morphology differed, a study in SW bulls found that sperm quality was affected by season although there was individual variation between bulls [4]. In Holstein bulls in northern Spain, sperm quality was best in spring, although sperm morphology and %DFI were not different among seasons [2]. A similar study on dairy bulls in Sweden indicated that %DFI was lowest in spring. In Thailand, effects of season on sperm quality were reported in different species. In swamp buffalo, %DFI was lower in the rainy season than in winter or summer, whereas MI and stability were higher in winter than in the rainy season or summer [16]. In boars, a seasonal effect was found on sperm morphology, sperm volume and total sperm production with high temperatures and high humidity having negative effects on sperm quality [17,18].

Factors such as environment, housing, age and breed have been shown to influence sperm quality [1,7,17]. Although the bulls were kept in an open barn in our study there was only a slight effect of season on sperm quality. There was a tendency for breed and age of bulls to affect sperm quality but these factors could not be included in the statistical model due to the small sample size. The lack of a difference in climate between summer and rainy season during sampling in the present study could explain why there was not a more obvious difference in sperm quality. Alternatively, it could indicate that the bulls are well adapted to their environment with very little effect of seasonal changes in climate on sperm quality. In our study, the THI was very similar in summer and the rainy season, differing only from winter. However, other studies in which THI has been linked to milk production or conception rates in dairy cows, have set the threshold at which heat stress occurs at a lower level than those calculated here. Therefore, our bulls would have been subjected to heat stress in all seasons unless they were accustomed to such conditions.

In experiment 2, SLC-selected samples showed higher normal morphology and a lower proportion with bent tails than uncentrifuged controls, in partial agreement with previous studies. Since SLC can improve the proportion of normal spermatozoa and remove head and tail defects [28], fertility may be improved compared to controls. Sperm kinematics were improved in the SLC samples compared to controls. Since sperm kinematics might be indicative of fertility *in vivo*, [29–31] the SLC-selected bull sperm samples might have better fertility than controls, since total motility, progressive motility or BCF may be predictive of high fertility, whereas VCL was previously linked with low fertility [32]. Certainly, SLC-selected stallion spermatozoa produced a higher pregnancy rate after AI than controls [33]. However, pregnancy rates were not investigated in our study. Previous studies have shown differences in quality between SLC-selected and unselected bull spermatozoa [12,4]. However, there are several differences between the studies: with regard to timing of SLC (after 24 h in [14], compared to immediately after semen collection in [12] and in the present study).

## CONCLUSION

Our findings showed that season had an effect on sperm morphology, with ejaculates collected in winter having a higher proportion of normal spermatozoa than in the rainy season or summer. No effect of season was found on sperm kinematics, sperm viability, %DFI or MMP. These results suggest that these bulls were well adapted to their location. However, there was considerable variation among individual bulls in %DFI, MMP and sperm viability. Furthermore, SLC had a positive effect on sperm morphology and sperm kinematics, which could be expected to influence fertility. Our results suggest that SLC could be used prior to cryopreservation regardless of season to enhance normal sperm morphology and sperm kinematics of thawed bull sperm samples without adversely affecting other parameters of sperm quality. Further studies are needed to investigate other factors involved e.g. breed of bull, age and different husbandry conditions.

## Figures and Tables

**Table 1:** LSMEAN±SEM of semen characteristics before freezing (n = 91) in 3 seasons

Sperm parameter	Summer (n = 26)	Rainy (n = 33)	Winter (n = 18)
Volume (mL)	7.5±0.7	6.7±0.8	6.1±0.8
Concentration (10^9^/mL)	1.2±0.2	1.4±0.1	1.1±0.1
pH	6.2±0.1^a^	5.8±0.1^b^	5.5±0.1^c^
MO (%)	54.8±4.7	51.2±5.4	39.0±5.3
Normal morphology (%)	60.7±4.9^a^	57.4±4.9^a^	70.1±4.9^b^
Acrosome defects1) (%)	3.4±1.9^ab^	3.6±1.9^a^	0.6±1.9^b^
Abnormal acrosomes (%)	15.6±2.9	18.8±2.9	16.3±2.9
Proximal droplet1) (%)	13.8±4.5^a^	10.5±4.5^ab^	3.4±4.5^b^
Detached head (%)	3.7±1.5	3.4±1.5	3.8±1.5
Bent tail (%)	5.0±2.9^ab^	7.9±2.9^a^	3.6±2.9^b^

LSMEAN±SEM, least squares means±standard error of the mean; MO, subjective motility.

1) Acrosome defects and Proximal droplet were treated with log transformation.

a–c Different superscript letters within a row indicate significant difference (p<0.05).

**Table 2:** Post-thaw sperm kinematics (n = 91), viability, DNA fragmentation index and plasma membrane integrity (n = 72) in bull semen in different seasons in Thailand (LSMEAN±SEM)

Sperm parameter	Summer (n = 26)	Rainy (n = 33)	Winter (n = 32)
MOT (%)	59.9±4.6	56.6±4.4	59.9±4.4
PRO (%)	22.7±2.9	19.8±2.8	19.5±3.0
SLOW (%)	15.3±1.5	14.9±1.5	17.0±1.5
STAT (%)	40.1±4.6	43.4±4.4	40.0±4.4
VAP (μm/s)	68.7±4.8	70.7±4.7	68.9±4.7
VCL (μm/s)	121.7±9.6	129.1±9.4	127.7±9.4
VSL (μm/s)	53.2±3.7	53.9±3.6	51.5±3.6
ALH (μm)	6.9±0.5	6.7±0.5	6.9±0.5
BCF (Hz)	23.8±0.8	24.6±0.8	24.3±0.8
LIN (%)	45.8±1.6	44.7±1.6	43.2±1.6
STR (%)	77.1±1.3	76.5±1.3	74.7±1.3
WOB (%)	58.0±1.1	56.9±1.1	56.3±1.1

LSMEAN±SEM, least squares means±standard error of the mean; MOT, total motility; PRO, progressive motility; SLOW, slow motility; STAT, statistic motility; VAP, velocity average path; VCL, velocity curved line; VSL, velocity straight line; ALH, amplitude of lateral head displacement; BCF, beat cross frequency; LIN, linearity; STR, straightness; WOB, wobble.

**Table 3:** Viability, DNA fragmentation index and plasma membrane integrity (n = 72) in bull semen in different seasons in Thailand (LSMEAN±SEM)

Sperm parameter	Summer (n = 24)	Rainy (n = 26)	Winter (n = 22)
Viability (%)	30.9±5.8	33.7±5.8	39.1±5.8
%DFI (%)	5.6±1.5	5.5±1.5	5.2±1.2
High MMP (%)	35.5±5.1	34.9±5.1	40.8±5.2

LSMEAN±SEM, least squares means±standard error of the mean; %DFI, DNA fragmentation index; MMP, mitochondria membrane potential.

**Table 4:** Comparison of post-thaw sperm morphology in bull semen between control and SLC selected sperm in Thailand (LSMEAN±SEM) (n = 54)

Sperm morphology	Control	SLC
Normal (%)	65.2±3.3^a^	68.6±3.3^b^
Acrosome defects (%)	2.6±1.5	2.6±1.5
Abnormal acrosomes (%)	16.9±1.4	15.9±1.4
Proximal droplet (%)	9.3±3.8	8.3±4.5
Detached head (%)	3.6±1.0	3.9±1.0
Bent tail (%)	5.5±2.2^a^	3.6±2.2^b^

SLC, single layer centrifugation; LSMEAN±SEM, least squares means±standard error of the mean.

a,b Different superscript letters within a row indicate significant difference (p<0.05).
